# Correction: The WHO atlas for female-genital schistosomiasis: Co-design of a practicable diagnostic guide, digital support and training

**DOI:** 10.1371/journal.pgph.0003403

**Published:** 2024-06-14

**Authors:** The PLOS GLOBAL PUBLIC HEALTH Staff

The affiliation for the second author is incorrect. The correct affiliation is not indicated. Pamela S. Mbabazi is not affiliated with #2 but with: Department of Control of Neglected Tropical Diseases, World Health Organisation, Geneva, Switzerland.

The publisher apologizes for the error.
