# Supplementary material for: The WHO atlas for female-genital schistosomiasis: Co-design of a practicable diagnostic guide, digital support and training
Source: PLOS Glob Public Health. 2024 Mar 18;4(3):e0002249. doi: 10.1371/journal.pgph.0002249 (PMC10947668; doi:10.1371/journal.pgph.0002249)
Supplement: Attachment — Submitted filename: Response to reviewers.docx [file pgph.0002249.s002.docx]

Response to Reviewers

*EC-1. Editor’s comment (EC):*

In the ethics statement in the Methods, you have specified that verbal consent was obtained. Please provide additional details regarding how this consent was

documented and witnessed, and state whether this was approved by the IRB.

*Response from the authors:*

Thank you for the comment. A detailed explanation of the ethic statement has now been included in the method section (Page 9).

*EC-2. Editor’s comment:*

Please amend your detailed online Financial Disclosure statement. This is published with the article. It must therefore be completed in full sentences and contain the exact wording you wish to be published.

a) State the initials, alongside each funding source, of each author to receive each grant. For example: "This work was supported by the National Institutes of Health (####### to AM; ###### to CJ) and the National Science Foundation (###### to AM)."
b) State what role the funders took in the study. If the funders had no role in your study, please state: “The funders had no role in study design, data collection and analysis, decision to publish, or preparation of the manuscript.”

*Response from the authors:*

The online financial disclosure statement has been updated, following the guidelines provided.

*EC-3. Editor’s comment:*

Please ensure that the funders and grant numbers match between the Financial Disclosure field and the Funding Information tab in your submission form. Note that the funders must be provided in the same order in both places as well.

*Response from the authors:*

The funders and grant numbers between the Financial Disclosure field and the Funding Information tab in the submission form have been double checked, and introduced in the same order.

*EC-4. Editor’s comment:*

Please update your online Competing Interests statement. If you have no competing interests to declare, please state: “The authors have declared that no competing interests exist.”

*Response from the authors:*

The online competing interests statement has been updated.

*EC-5.* *Editor’s comment:*

Please ensure that you refer to Figure 7 in your text as, if accepted, production will need this reference to link the reader to the figure.

*Response from the authors:*

The previous caption of Figure 7 has been removed from the text. The previous Figure 8 is now Figure 7. There were only 7 Figures included in the manuscript. The Figure number is now corrected and referred in the text.

*EC-6.* *Editor’s comment:*

Please include a legend for Figure 7 in your manuscript.

*Response from the authors:*

There were only 7 Figures in the manuscript. The previous Figure 8 is now Figure 7. The Figure number is now corrected and referred in the text.

*EC-7.* *Editor’s comment:*

Please ensure that you refer to Tables 3, 4 and 5 in your text as, if accepted, production will need these references to link the reader to the tables.

*Response from the authors:*

References to Tables 3, 4 and 5 have been included in the text.

*EC-8.* *Editor’s comment:*

Some material included in your submission may be copyrighted. According to PLOS’s copyright policy, authors who use figures or other material (e.g., graphics, clipart, maps) from another author or copyright holder must demonstrate or obtain permission to publish this material under the Creative Commons Attribution 4.0 International (CC BY 4.0) License used by PLOS journals. Please closely review the details of PLOS’s copyright requirements here: PLOS Licenses and Copyright. If you need to request permissions from a copyright holder, you may use PLOS's Copyright Content Permission form.

Please respond directly to this email or email the journal office and provide any known details concerning your material's license terms and permissions required for reuse, even if you have not yet obtained copyright permissions or are unsure of your material's copyright compatibility.

*Response from the authors:*

The images corresponding to the Figures 1, 2, 3, 4 and 8 have been modified, removing all the logos and any other element that may produce copyright infringement of the PLS’s copyright policy.

*EC-9.* *Editor’s comment:*

Potential Copyright Issues: Figures 1, 2, 3, 4 and 8 includes an image of an identifiable person. Please provide written confirmation or release forms, signed by the subject(s) (or their parent/legally authorized guardian), giving permission to be photographed and to have their images published under our CC-BY 4.0 license.

Otherwise, we kindly request that you remove the photograph.
Figures 4 and 8 contains branding/a logo. We are not permitted to publish this under our CC-BY 4.0 license, even with permission. We ask that you please remove or replace it.

*Response from the authors:*

Figures 1, 2, 3, 4 and 8 have been modified to make the people unidentifiable.

*EC-10.* *Editor’s comment:*

Please review your reference list to ensure that it is complete and correct. If you have cited papers that have been retracted, please include the rationale for doing so in the manuscript text, or remove these references and replace them with relevant current references. Any changes to the reference list should be mentioned in the rebuttal letter that accompanies your revised manuscript. If you need to cite a retracted article, indicate the article’s retracted status in the References list and also include a citation and full reference for the retraction notice.

*Response from the authors:*

The reference list has been double checked to ensure that it is complete and correct.

*R1-1.* *Reviewer’s comment:*

It would be interesting to know within the manuscript how many participants had colposcopy performed and how many images were originally obtained in order to select representative samples (the atlas itself suggests 10,000 but this is not discussed within the manuscript).

*Response from the authors:*

A description of the total number of images (10,000) has been now included in the text (page 16).

*R1-2.* *Reviewer’s comment:*

The first of the workshops was held on 04/10/2014, designing the atlas (WHO published in 2015). As it is now 2023, I feel the authors should discuss (or at least acknowledge) the extensive time-lag in publishing these data. Since it has been several years – do the authors have any data on the implementation of the atlas +/- the corresponding poster (note the lack of data would not preclude publication)?

*Response from the authors:*

A justification for the time-lag in publishing the data has been included in the discussion. In addition, several reflections on the implementation of the atlas and the corresponding paper are also written (page 26).

*R1-3.* *Reviewer’s comment:*

It would be useful to include the web address of the WHO atlas download page:

Female genital schistosomiasis: A pocket atlas for clinical health-care professionals (who.int)

*Response from the authors:*

The full web address for the WHO atlas has been included int the references (<https://apps.who.int/iris/rest/bitstreams/812164/retrieve>).

*R1-4.* *Reviewer’s comment:*

Page 13 first sentence: “Before the workshop, two FGS experts (EFK and HGA) sorted images by lesion type and quality and selected 28 images were chosen for each of the four FGS lesion types.” This sentence is ambiguous as it seems to be reporting 28 images were pre-selected (but I’m aware only 28 images were included in the final atlas).

*Response from the authors:*

A description of the selection of images, providing its criteria, has now been included in the manuscript (page 16).

*R1-5.* *Reviewer’s comment:*

Page 15 second sentence: “The experts had pre-selected 60 images of the types of lesions of FGS.” Please clarify how many images were pre-selected.

*Response from the authors:*

A description of the pre-selection of images, providing its criteria, has now been included in the manuscript (page 16).

*R1-6.* *Reviewer’s comment:*

Table 3 page 20: It is not clear what 'Categories 1 and 2' represent? Please include a description in the table and/or legend.

*Response from the authors:*

Considering this comment and the subsequent one, the tables have been rewritten to show the summary of the content. The division of the content analysis by HCD phases has been merged to ease the reading of the table, focusing on the summary of the content analysis. There was no content analysis in the atlas design workshop. Therefore no new table is included.

*R1-7.* *Reviewer’s comment:*

Table 5 page 27: Please state what the 2x question marks (?) along with the 'Category' represent. It may be useful to include a content analysis summary table with HCD-phases for the first workshop, atlas design. It would also be clearer to have content analysis summary tables displaying consistent column headings e.g., HCD Phase (column heading on Table 3 but not 4).

*Response from the authors:*

The symbols have been removed, and the headings are now consistent across the tables.

*R2-1.* *Reviewer’s comment:*

Data availability. Please clearly write the data availability statement

*Response from the authors:*

A data availability statement has been written. The research team has applied for funding to make a database in liaison with the WHO.

*R2-2.* *Reviewer’s comment:*

Female genital schistosomiasis (FGS) is a significant health problem that affects up to 56 million females globally. The number refers to African origin in the abstract but is now referred to internationally. Please clarify and provide a reference.

*Response from the authors:*

The number refers to African origin in the abstract but is now referred to internationally. Please clarify and provide a reference.

*R2-3.* *Reviewer’s comment:*

The parasitic disease is acquired through contact with contaminated freshwater bodies used for daily chores and livelihoods, particularly among women and girls in rural communities. Could you please be more descriptive about the regions and the context's history? This will assist the reader in comprehending the context.

*Response from the authors:*

A reference to the rural or semi-urban communities in Africa has been included in the text (page 4).

*R2-4.* *Reviewer’s comment:*

In these workshops we sought to provide a medical booklet and bedside poster with examples of FGS lesions, explore the requirements for a diagnostic support for FGS and eLearning for health professionals. Are you only referring to formal health professionals? If so, please specify.  As in rural areas, there are informal health care professionals who are easily accessible to members of the community. (Description on page 11)

*Response from the authors:*

An explicit mention to formal healthcare professionals has been written in the manuscript

*R2-5.* *Reviewer’s comment:*

Study information was provided to the target populations in the local languages for the image collection. Please describe the target demographics. Are the target populations of the four nations similar?

*Response from the authors:*

The target demographics have been described in the text (page 9).

*R2-6.* *Reviewer’s comment:*

"all women who fulfilled the inclusion

criteria were offered gynaecological examination by health professionals with expertise in FGS". Please state the inclusion criteria.

*Response from the authors:*

The inclusion criteria has been stated in the manuscript (page 9)

*R2-7.* *Reviewer’s comment:*

Three workshops were held on FGS. There are four countries, however only three workshops are mentioned. Please include the name of the nation where the workshops were held. Please also include the rationale for not doing a session in one country.

*Response from the authors:*

The images were collected in three countries. The HCD workshops were held in South Africa. These aspects have been now written in the manuscript (p. 10).

*R2-8.* *Reviewer’s comment:*

Any difficulties in photographing FGS symptoms, and how did you overcome them? A few lines will assist the reader to grasp the contexts of data acquisition.

*Response from the authors:*

The challenges photographing the FGS symptoms have been now described in the manuscript, together with a comprehensive description of the context of data acquisition. In addition, an explanation of how these were overcome has been also included (page 10).

*R2-9.* *Reviewer’s comment:*

"In 2019 and 2020, a total of 45 (18+27) participants attended two co-design workshops.” Could you please mention the third workshop?

*Response from the authors:*

The third workshop has been mentioned in the manuscript (page ).

*R2-10.* *Reviewer’s comment:*

Each group reviewed two disease manifestations (30 images, see Fig 1), selected the most appropriate ones based on predefined criteria of clarity and representativity,

*Response from the authors:*

The criteria for the review of the disease manifestation has been included in the manuscript (page 16).

*R2-11.* *Reviewer’s comment:*

"Telemedicine should be then part of the design to perform quality control and provide online assistance from a knowledgeable gynaecologist/trained person at the other end. A system should be in place to ensure patients are notified about their results." Please describe how this will work in low-resource settings.

*Response from the authors:*

A description of how the telemedicine would work in a low-resource setting has now been included in the paper (page 19).

*R2-12.* *Reviewer’s comment:*

Patient information and informed consent. This is a really important topic to tackle. I will request that the consent process be explained in detail since it should be transparent and culturally appropriate, and any language or cultural difficulties should be addressed.  Also, how to make it easier on the participants in terms of confidentiality. Many vulnerable women/girls agreed to consent because they desperately needed the service, but they later refused to share their private parts picture. As a result, their ability to refuse or withdraw consent at any time should be included in the pocket Atlas.

*Response from the authors:*

The content process has now been explained in detail, in order highlight its transparency and cultural appropriateness. In addition, a reference to confidentiality has also been included in the manuscript. An explicit mention to the ability of the participants to refuse or withdraw consent at any time has also been included in the manuscript (page 8).

*R2-12.* *Reviewer’s comment:*

"Schistosoma PCR should be done"

*Response from the authors:*

PCR has been written as polymerase chain reaction in the text (p. 26). MDA was already written mass drug administration the first time mentioned in the text (p. 29)

PZQ has been written as praziquantel (p. 34).
