# Supplementary material for: The WHO atlas for female-genital schistosomiasis: Co-design of a practicable diagnostic guide, digital support and training
Source: PLOS Glob Public Health. 2024 Mar 18;4(3):e0002249. doi: 10.1371/journal.pgph.0002249 (PMC10947668; doi:10.1371/journal.pgph.0002249)
Supplement: Attachment — Submitted filename: Response to reviewers.docx [file pgph.0002249.s005.docx]

Response to Reviewers

*R1-1.* *Reviewer’s comment:*

Please clarify who participated in the workshops (e.g., health professionals, researchers, patients, etc.)

*Response from the authors:*

Thank you for the comment. The participants’ categories have been included in the abstract

*R1-2.* *Reviewer’s comment:*

In the introduction, co-design method has been introduced really well with brief explanation covering its definition and scope, please provide some justification for why a co-design approach was chosen over other methods for developing these tools or else can be cross referenced with the citation below where it justifies being implemented for school-aged children in Zanzibar.

*Response from the authors:*

Thank you for the comment. A justification for the use of co-design methods has been included, also cross- referencing the paper that described the intervention in Zanzibar.

*R1-3.* *Reviewer’s comment:*

Avoid labeling participants as "not virgins" as this could come across as stigmatizing. Instead, you could say the study included sexually active adult women within a certain age range.

*Response from the authors:*

Thank you for the comment. “Not virgins” has been removed. A reference to the study population was already included in the previous version ans it is maintained: “In all countries target populations were sexually active women of reproductive age only”

*R1-4.* *Reviewer’s comment:*

Specify how the initial informed consent process was conducted (e.g. written vs. verbal, language used, etc.) and by whom. State that consent was re-confirmed verbally before each procedure rather than "reascertained."

*Response from the authors:*

Thank you for the comment. The specification of the informed consent process has been included in teh text. The initial informed consent was written except in Zimbabwe where it was verbal. «Re-ascertained» has been replaced by "re-confirmed".

*R1-5.* *Reviewer’s comment:*

Note that participants were informed they could withdraw consent at any time without penalty.

*Response from the authors:*

Thank you for the comment. It has been included in the manuscript.

*R1-6.* *Reviewer’s comment:*

Clarify that the IRB or ethics board approved the overall study procedures and consent process, even if they didn't review each individual consent form.

*Response from the authors:*

Thank you very much for the comment. The clarification has been included (page 9).

*R1-7.* *Reviewer’s comment:*

Remove any justification for why certain patients were excluded (e.g. not seriously ill). Focus on who was included rather than who was excluded.

*Response from the authors:*

Thank you for the comment. The sentence has been removed.

*R1-8.* *Reviewer’s comment:*

Specify the dates or timeframe when the images were collected.

*Response from the authors:*

Thank you for the comment. The following text has been added to the manuscript (“Photocolposcopic images were captured from women, between 1994 and 2012 in four different study sites endemic for S. haematobium in Malawi, Zimbabwe, South Africa and Madagascar. Images and specimens were sampled from sexually active women between 15 and 49 years of age. Colposcopic images of other diseases are included for differential diagnostic purposes. Nortseth et al., 2014)

*R1-9.* *Reviewer’s comment:*

Explain why three countries were chosen as data collection sites. Were the settings and populations similar or intentionally varied?

*Response from the authors:*

Thank you for the comment. The following text has been added in the manuscript: "They were intentionally different in terms of age and endemicity"

*R1-10.* *Reviewer’s comment:*

Consider mentioning any challenges faced in obtaining quality images and how these were addressed.

*Response from the authors:*

Thank you for the comment. A detailed explanation has been included (page 10).

*R1-11.* *Reviewer’s comment:*

For the workshops, clarify the locations in South Africa where they took place.

*Response from the authors:*

Thank you for the comment. The information has been added.

*R1-12.* *Reviewer’s comment:*

State how many participants attended each workshop and how they were selected/recruited.

*Response from the authors:*

Thank you for the comment. The information has been added.

*R1-13.* *Reviewer’s comment:*

Provide a bit more detail on how participants were recruited and enrolled. Were there any inclusion/exclusion criteria?

*Response from the authors:*

Thank you for the comment. The information has been added.

*R1-14.* *Reviewer’s comment:*

Consider organizing the definitions into logical categories (e.g. disease characteristics, clinical procedures, health provider roles). And make sure the terms being defined here are consistently applied throughout the manuscript.

*Response from the authors:*

Thank you very much for the comment. The suggestions have been implemented.

*R1-15.* *Reviewer’s comment:*

Consider organizing the definitions into logical categories (e.g. disease characteristics, clinical procedures, health provider roles). And make sure the terms being defined here are consistently applied throughout the manuscript.

*Response from the authors:*

Thank you for the comment. The categories have been introduced. These sentences are considered relevant by the authors because they were part of the diagnostic practical standard being developed in the WHO FGS pocket Atlas. In the case of the speculum, it is relevant for the connection with the training and education addressed in the second and third workshops.

*R1-16.* *Reviewer’s comment:*

For brand name instruments like the Olympus colposcope, follow with (City, State/Country) after the first mention.

*Response from the authors:*

Thank you for the comment. The format has been followed

*R1-17.* *Reviewer’s comment:*

Make sure to use the proper trademarks and register symbols (® TM) for brand/product names.

*Response from the authors:*

Thank you for the comment. The first version of the manuscript included them, but the journal’s editors asked to be removed following the guidelines of the journal

*R1-18.* *Reviewer’s comment:*

12. Specify the camera models used with the Leisegang colposcope.

picture. As a result, their ability to refuse or withdraw consent at any time should be included in the pocket Atlas.

*Response from the authors:*

Thank you for the comment. The information has been written on page 14.

*R1-19.* *Reviewer’s comment:*

Consider mentioning the stationery supplies in the methods where workshops are described rather than the materials section. Also check that all materials mentioned here are referred to in the methods where they were utilized.

*Response from the authors:*

Thank you for the comment. The text referring stationary supplies have been moved to the methods section where they were utilised

*R1-20.* *Reviewer’s comment:*

For audiovisual recordings, specify if it was video, voice recordings, photographs etc.

*Response from the authors:*

Thank you for the comment. The specification has been included on page 14.

*R1-21.* *Reviewer’s comment:*

For the image analysis, clarify how many images were originally collected and how the process narrowed down to 28 final images.

*Response from the authors:*

Thank you for the comment. The amount of images (10 000) has been specified together with a more detailed explanation of the selection procedure.

Entered in the dataset. Found positive cases (registered having FGS), amongst those we sort out particular images and looked for images that were of good quality observable lesions.

*R1-22.* *Reviewer’s comment:*

For workshop #2 and #3, provide a bit more detail on the qualitative analysis process - how were transcripts analyzed? What codes/themes emerged?

*Response from the authors:*

Thank you for the comment. The details have been provided and indicated where they are placed.

*R1-23.* *Reviewer’s comment:*

Consider restructuring the agreed outcomes for the 3 workshops into logical subsections rather than jumping between topics.

*Response from the authors:*

Thank you very much for the comment. The outcomes have been restructured into logical subsections/paragraphs in the results section.

*R1-24.* *Reviewer’s comment:*

Avoid repetition between sections - for example the participants and setting are described twice for workshop #3.

*Response from the authors:*

Thank you for the comment. The repetition have been fixed.

*R1-25.* *Reviewer’s comment:*

Consider adding a limitations section on the data analysis approach.

*Response from the authors:*

Thank you very much for the comment. A research limitations sections has been included, addressing the data analysis approach.

*R1-26.* *Reviewer’s comment:*

Please mention this number above.

*Response from the authors:*

Thank you for the comment. The number is now mentioned.

*R1-27.* *Reviewer’s comment:*

The explanation of how telemedicine would work seems quite brief still. Can you expand on the technical details of how images/data would be transmitted and stored? How would quality control and online assistance actually be implemented?

*Response from the authors:*

Thank you very much for the comment. A brief explanation is given in the text (page  19), describing ongoing efforts to implement such a system in the current research project (funded by the European Union). Details that could be commercially sensitive are omitted.

*R1-28.* *Reviewer’s comment:*

The expanded description of the consent process is helpful. One aspect not explicitly mentioned is the ability of participants to withdraw consent after the fact - i.e. can they request their images be withdrawn/destroyed if they later change their mind? This could be worth clarifying**.**

*Response from the authors:*

Thank you for the comment. Yes, this was already mentioned on page 8 (“in the consent procedures, the participants were informed that they could withdraw their consent at any time without penalty and that they could request their material (e.g., "images to be consequently destroyed. ”)

*R1-29.* *Reviewer’s comment:*

Expand on how nurses and other healthcare workers will be trained on FGS diagnosis. Consider providing more specifics on the proposed eLearning course curriculum and format.

*Response from the authors:*

Thank you for the comment. These have been added on page 23.

*R1-30.* *Reviewer’s comment:*

*For the graduate training, discuss whether FGS could be incorporated into clinical rotations or hands-on training with models/mannequins.*

*Response from the authors:*

Thank you for the comment. These have been added on page 26.

*R1-31.* *Reviewer’s comment:*

Address any potential barriers to implementation of the awareness and training initiatives discussed. For example, costs, accessibility in remote regions, cultural factors etc.

*Response from the authors:*

Thank you for the comment. Briefly mentioned on page 26.

*R1-32.* *Reviewer’s comment:*

When discussing community leaders, consider their specific role in treatment programs - will they help encourage participation? Monitor for side effects?

*Response from the authors:*

Thank you for the comment. Added on page 28.

*R1-33.* *Reviewer’s comment:*

Discuss plans for evaluating the impact of the proposed training and awareness programs. How will you assess their effectiveness?

*Response from the authors:*

Thank you for the comment. Briefly mentioned on page 26. It is also part of the ongoing research project.
